# A Temporal Signal-Processing Circuit Based on Spiking Neuron and Synaptic Learning

**DOI:** 10.3389/fncom.2019.00041

**Published:** 2019-06-28

**Authors:** Hui Wei, Yi-Fan Du

**Affiliations:** Laboratory of Cognitive Model and Algorithm, Shanghai Key Laboratory of Data Science, Department of Computer Science, Fudan University, Shanghai, China

**Keywords:** time-related neuron, time-processing circuit, spiking-neuron, synaptic learning, ramp activity, SCT

## Abstract

Time is a continuous, homogeneous, one-way, and independent signal that cannot be modified by human will. The mechanism of how the brain processes temporal information remains elusive. According to previous work, time-keeping in medial premotor cortex (MPC) is governed by four kinds of ramp cell populations (Merchant et al., 2011). We believe that these cell populations participate in temporal information processing in MPC. Hence, in this the present study, we present a model that uses spiking neuron, including these cell populations, to construct a complete circuit for temporal processing. By combining the time-adaptive drift-diffusion model (TDDM) with the transmission of impulse information between neurons, this new model is able to successfully reproduce the result of synchronization-continuation tapping task (SCT). We also discovered that the neurons that we used exhibited some of the firing properties of time-related neurons detected by electrophysiological experiments in other studies. Therefore, we believe that our model reflects many of the physiological of neural circuits in the biological brain and can explain some of the phenomena in the temporal-perception process.

## Introduction

When we use visual cues to observe the environment, we need to grasp both the time interval and the sequence of various events. When we wish to understand speech, we need to distinguish between the arrival times of the audio signals. We need to accurately control the order of execution of motor commands to skeletal muscle to perform activities such as speaking and playing the piano. When we solve problems, we also need to plan the chronological order of all sub-goals. These frequent daily tasks indicate that the capacity for temporal information processing, like other cognitive abilities such as working memory, must be one of the basic functions of the brain. Clearly, humans can perceive a broad spectrum of time scales. At present, the neurocognitive community usually divides the temporal-processing range of the brain into four categories: microsecond-scale processing, millisecond-scale processing, second-to-minutes-scale processing, and circadian-rhythm processing (Merchant and Lafuente, 2014). In our work, we focus on millisecond-scale processing. The processing of millisecond-scale timing information is the most common, and it is usually accompanied by various types of sensing and motor control. Most research on millisecond-scale processing concerns motion control and auditory time perception. Temporal processing in the hundreds of milliseconds is quite sophisticated. And its neural underpinnings are largely unknown yet. In a study on motion control, researchers discovered that neurons in the motor cortex convey information via spike timing far more often than via spike rate (Tang et al., 2014). In addition, they found that the amount of information conveyed at the millisecond timescale greatly exceeds the information available from spike counts. The findings of this important study have guided our time-perception model.

There are many tasks used to study perceptual and motor timing. Perceptual timing is considered as a subjective judgement of perceived timing and is not defined by movement. However, motor timing is a kind of temporal process where the temporal decision is intrinsically tied with movement. Hence, perceptual and motor tasks represent two different kinds of tasks used to study temporary-processing mechanisms in our brain. Classic motor and perceptual timing tasks have been summarized previously (Merchant and Lafuente, 2014). The task applied in our work, called the synchronization-continuation tapping task (SCT), is a kind of motor timing task.

In the current paper, we present a complex network in order to simulate the synchronization-continuation experiment with as much biological feasibility as possible. Here, we designed our model from the perspective of structure just like the work we used to do (Wei et al., 2017; Hui and Dawei, 2018). We considered not only complex topologies, but also synaptic plasticity in order to determine the time interval. And via the considered structure, we were able to simulate the SCT experiment and obtain the spiking neuron with a firing rate similar to that observed in electrophysiological experiments.

### Synchronization-Continuation Tapping Task

**Figure 1:**
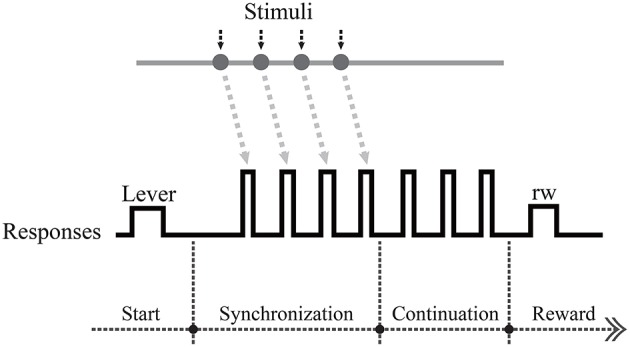
Schematic overview of intervals in the synchronization-continuation tapping task (SCT), showing periodic stimuli (gray line), and push button responses (black line). Each trial began when the monkey held a lever.

In this study, the model we employed was based on experiments on the millisecond scale. A recent study found that the activity of cells in the medial prefrontal cortex (MPC) of macaques could characterize the time course of SCT experiments (Merchant et al., 2014). In an SCT experiment, the subject first responds synchronously with a visual or auditory metronome and then continues to produce the same interval without the metronome (Figure 1). In addition, they tested the neurophysiological properties of two macaque MPCs in the SCT experiment and found that the timing function of the MPC is determined by different cell populations. These researchers proposed four different types of neurons, which they labeled as swing cells, relative-timing cells, absolute-timing cells, and time-accumulator cells. During the SCT experiment, these neuronal types were discovered to display different forms of ramp activity, which encodes the elapsed time since the last motion or the remaining time until the next tap. This experiment showed that the MPC has a mechanism for the time-correlated analysis of rhythmic, time-series, and repetitive signals. This was a sub-second task. It remains to be elucidated what kind of neural circuit can acquire the time interval during the synchronization phase and repeat the action at this time frequency in the continuous phase.

There are several neurophysiological underpinnings of beat-based timing during SCT investigations (Merchant and Bartolo, 2018). In addition to the ramp activity in SCT mentioned above (Merchant et al., 2011), another study found that MPC neuronal populations dynamically represent the duration and serial order during the SCT (Crowe et al., 2014). It has also been found that there is tuning for interval and/or serial order as an orderly change in the power of transient modulations in β- and γ- bands across putaminal LFPs during the execution of the SCT (Bartolo et al., 2014). Recent research shows that the neural population trajectories during SCT in SMA/preSMA can act as a neural clock (Gámez et al., 2019). However, the neural code linked to the temporal production of this neural clock during SCT remains unknown. Moreover, elucidating the neural underpinnings of motor timing is critical to understanding how sensorimotor systems can predict the regular pulse and then respond with temporal precision.

The two goals of the current paper are as follows: (1) to establish a network circuit model of spiking neuron to simulate pyramidal cells and interneurons in order to achieve the time interval learning of the synchronous phase and the spontaneous follow-up function of the continuous phase in the SCT experiment; and (2) to simulate the SCT experiment in macaques by implementing the ramp activity of the four different neuron types proposed previously by Merchant et al. (2011).

### Ramp Activity

One of the most important tasks of the brain is to anticipate upcoming events in order to prepare for behavior, anticipate reactions, and plan. The phenomenon of ramping firing rates prior to behavioral responses is commonly observed in behavioral neuroscience, and—in many cases—is anticipatory in nature.

Ramp activity, which can be defined as delayed activity that steadily increases between two subsequent stimuli, has been associated with the anticipation of various events, such as motor responses (Constantinidis and Steinmetz, 1996), the end of the delay interval (Romo et al., 1999; Reutimann et al., 2001), or the identity of the sample or match stimulus in delayed matching-to-sample (DMS) tasks [retrospective vs. prospective coding (Rainer et al., 1999; Mongillo et al., 2003)]. The increasing delayed activity can also be associated with reward expectation, such as that found in the prefrontal cortex (Watanabe, 1996), striatum (Kawagoe et al., 1998; Hassani et al., 2001), thalamus (for review, see Schultz, 2000; Komura et al., 2001), and motor cortex (Merchant et al., 2004). Some experiments have addressed the question of how a change in the duration of the delay period is reflected in the time-varying delay activity (Kojima and Goldman-Rakic, 1982; Komura et al., 2001; Brody et al., 2003). In these experiments the build-up of activity is stretched in time, rather than shifted. The stretching causes the slope of the activity profile to decrease with the length of the delay period. This is in agreement with the scaling property of interval timing found in psychophysical studies on humans (Rakitin et al., 1998) and has recently confirmed by *in vivo* experiments in monkeys (Leon and Shadlen, 2003). When the duration of a time interval is estimated, the error distribution scales linearly with the length of the interval.

Prediction requires animals to extract and exploit the temporal structure of their world, or the temporal relationship between environmental events or their own behavior and associated effects. Prediction is seen as a symbol of expectation in time-perception tasks. Ramp activity, sometimes referred to as climbing activity, is considered to be prospective. Recordings from different areas in the cortices of monkeys suggest the existence of neurons representing time by ramp (climbing) activity, which is triggered by an initial event and peaks at the expected time of a second event, such as a visual stimulus and a reward. The activity of this neuron is often a good indicator of the duration of the two events. In studies provided by Leon and Shadlen (2003), we see that different slopes of climbing activity can be used to calibrate different time intervals. This not only reveals that the slope of neuronal climbing activity can be used to characterize time, also that different time intervals can be learned by determining the slope of the climbing activity.

### Structure of This Paper

The remainder of this paper details our research as follows: related works are presented in section Related Work. Section A Spiking-Neuron Circuit For Temporal Signal-Processing describes our spiking-neuron circuit for temporal signal-processing and the synaptic learning algorithm we used. In this section, we also introduce the structure of our neural circuit in detail. In section Computational Simulation Results, we compare the simulation results of our computational model with biological results found in SCT experiments from previous studies and explain some electrophysiological phenomena. Finally, we present conclusions and discuss our research in section Conclusion and Discussion.

## Related Work

There are many computational models for time-dependent signal processing, including pacemaker accumulator models (Treisman, 1963), state dependent network models (Buonomano and Maass, 2009), long short-term memory models(LSTM) (Rivest et al., 2010), time-adaptive drift–diffusion models (TDDM) (Rivest and Bengio, 2011), and recurrent synaptic networks (Mendoza et al., 2018).

The pacemaker accumulator model is a traditional time model proposed many years ago (Treisman, 1963), the concept of which was derived from mechanical clocks. This model assumes that there is a pacemaker or an oscillator in our brain that sends pulses consistently at a certain frequency, and these are received and recorded by an accumulator. Within this framework, the pulse count provides a linear metric of time, and temporal judgments rely on comparing the current pulse count with that of a reference time. This process becomes the foundation for characterizing time in this model. The pacemaker accumulator model has proven to be effective in providing a framework for many psychophysical data related to time processing (Church, 1984; Meck, 2005). The downside of this model, however, is that it lacks biological feasibility. Mounting evidence indicates that clock models are not entirely consistent with the experimental data (for reviews see Mauk and Buonomano, 2004; Buhusi et al., 2005).

The state-dependent network model recently proposed by Buonomano et al. differs from these above models. This model is able to tell and encode time as a result of dynamic change in the state of spiking neural networks. It is based on the assumption that there is an interaction between each sensory event and the current state of the network, forming a network state pattern that naturally encodes each event in the context of recent stimuli—similar to the interaction between different ripples generated by each raindrop falling in a pond instantly or previously. State-dependent models have the powerful ability to characterize time since they are inherently high dimensional. However, the deficiency of this model is that it encodes time via the firing rate of each neuron in the model, which is contrary to the result of Buonomano's motor-control experiment, in which the spiking time conveyed more information than the spiking rate [millisecond-scale motor encoding in a cortical vocal area].

In addition, LSTM and temporary difference learning (TD) algorithms have been used to propose a small neural network based on artificial neurons that can encode a specific time into a ramp-like activity (Rivest et al., 2010). Although they introduced many biological concepts into their model, the basis of the model is the artificial neuron which is far from the bioneuron compared to the spiking neuron.

TDDM was independently proposed by Rivest and Bengio (2011) and Simen et al. (2011) which utilizes a simple and more abstract neural model based on a drift-diffusion process of climbing neural activity. The drift-diffusion model is often used in decision-making under noisy stimuli. This work extends it by developing a learning rule so that their model can be used to learn time intervals rapidly. Additionally, Weber's law for time can be explained in this study.

There is another excellent model. Recently, a kind of model called a recurrent synaptic network has been proposed (Mendoza et al., 2018). It simulates a cortical ensemble and makes use of paired-pulse facilitation and slow inhibitory synaptic currents to not only produce interval selective responses but also to follow the biases and scalar properties (Pérez and Merchant, 2018).

In addition to the millisecond-range time-processing model mentioned above, there are several time-processing models in seconds to minutes range such as striatal beat frequency model (SBF), which is proposed by Matell and Meck (2004). SBF suggests that in the thalamo-cortico-striatal loops, the coincidence detection of neuronal oscillations in the cortex is the neural basis for the characterization of time information. Cortical neurons will act as oscillators and the striatum located in the basal ganglia can detect the oscillation pattern of cortical neurons. At the beginning of time interval processing, the release of dopamine in the brain prompts timing and synchronizes cortical oscillations, and resets the state level of striatum spinous neurons. The cortical oscillators oscillate a fixed frequency throughout the criterion interval. At the end of time interval processing, dopamine is released again, which changes the synaptic connections of spinous neurons, and forms the neural representation of time interval.

## A Spiking-Neuron Circuit for Temporal Signal-Processing

### Neuron Model

In this paper, a simple spiking neuron model known as the Izhikevich neuron model was used to simplify the Hodgkin–Huxley (HH) model into a 2-D system with sufficient biological plausibility and high computational efficiency (Izhikevich, 2003). The form of the ordinary differential equation is shown in Equation 1 where V represents the membrane potential of the neuron, u represents a membrane recovery variable, and a, b, c, and d are dimensionless parameters. We have the following:

(1) $$\begin{array}{l} \;\frac{dV}{dt}=0.04V^{2}+5V+140-u+I\\ \;\frac{du}{dt}=\text{a}\left(\text{bV}-\text{u}\right)\\ \text{If V}\geq 30,\text{ then }\{\begin{array}{c} V\;\leftarrow c\\ u\;\leftarrow u+d \end{array} \end{array}$$

**Figure 2:**
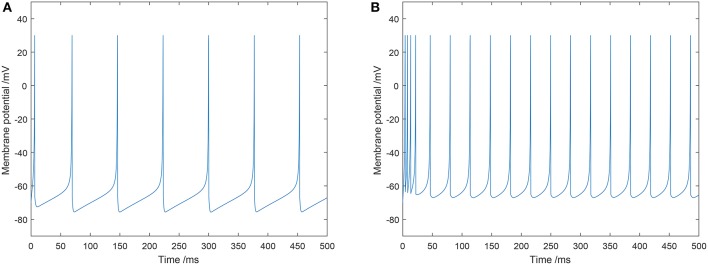
Two types of spiking patterns: **(A)** regular spiking for excitatory neurons; **(B)** fast spiking for inhibitory neurons.

In this study, typical values of the parameters for an excitatory neuron were: *a* = 0.02, *b* = 0.2, *c* = −65, and *d* = 8. Typical values of the parameters for an inhibitory neuron were: *a* = 0.1, *b* = 0.2, *c* = −65, and *d* = 2. The firing mode of the excitatory and inhibitory neurons we utilized in our model are shown in Figure 2.

In order to adequately describe the relationship between the firing rate of neurons and the time interval, we also introduce another kind of neuron model, as shown in the following equation (the equation from Gavornik et al., 2009).

(2) $$\begin{array}{r} \gamma_{m}\frac{dV_{i}}{dt}=-V_{i}+I_{ext,i}+\overset{N}{\underset{j=1}{\sum }}L_{ij}V_{j} \end{array}$$

In the equation, the firing rate of the single neuron j is approximated by an activity variable V, γ_*m*_ is an intrinsic neuronal time constant, *I*_*ext, i*_is the external feed-forward input to neuron i and *L*_*ij*_ is the weight connecting the presynaptic cell j to the postsynaptic cell i.

### Time-Adaptive Drift-Diffusion Models (TDDM)

In order to realize time adaptation, we referred to time-adaptive drift-diffusion models (TDDM). This model is also used to reflect the ability to learn the timing of events, but it is a simpler and more abstract neural model. TDDM takes advantage of the drift-diffusion model, commonly used in decision simulations, to encode specific time intervals by accumulating evidence of elapsed time with the drift rate (Rivest and Bengio, 2011). In TDDM, the memory of the time interval to be learned is stored in the drift rate, so that it can control the signal's slope as time elapses. This signal changes over time in a form very similar to the ramp activity observed in the MPC of macaques. Therefore, we believe that TDDM can be used to simulate the ramp activity of some neurons in the MPC, which can be used as our model's main learning interval mechanism for synaptic learning algorithms.

In the TDDM implementation process, the semaphore ϕ (t) is 0 at the beginning of the stimulus, and continuously accumulates as time passes. The overall process is similar to an accumulator, which integrates continuously over time with a drift rate w and noise ε(t). The main function of the model is expressed in the following form, which is similar to the drift-diffusion model:

(3) $$\begin{array}{r} \phi \left(\text{t}\right)=\phi \left(\text{t}-1\right)+\text{w}\Delta \text{t}+\epsilon \left(\text{t}\right) \end{array}$$

Where Δt is the time step and ε(t) is the Gaussian noise with a mean value of 0 and variance σ^2^ [N(0, σ^2^]. It is also stipulated that when the amount of information reaches its peak, a certain reward will be given, leading to the renewal of the drift rate. By constantly updating w through the experiments, our information volume can reach one near the target interval. Obviously, the learning process of this model involves two situations (as shown below).

As mentioned previously, ramp activity is considered to be prospective and can be used to express expectations of upcoming events. Here we use reward to represent the upcoming event, while the moment the semaphore ϕ(t) reaches one is called expected.

**Figure 3:**
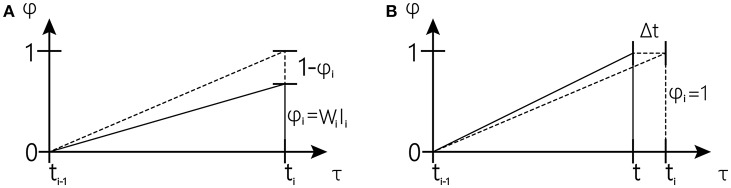
Schematic overview of one tapping interval in the synchronization-continuation tapping task (SCT), showing periodic stimuli (vertical dashed lines), and preparation signal for push button responses (vertical solid lines).The inclined dash line represents the desired trajectory; The inclined solid line represents actual trajectory (The figure is modified from Rivest and Bengio, 2011).

In the first cases (shown in Figure 3A), reward occurs earlier than expected, and the drift rate w toward the observed interval can be corrected at once using Equations (4) and (6) to increase the slope of the accumulator when reward occurs. We have the following:

(4) $$\begin{array}{r} \Delta \text{w}\left(\text{n}\right)=\text{w}\left(\text{n}\right)\frac{\left(1-\varphi \left(t\right)\right)}{\varphi \left(t\right)} \end{array}$$

In the second case (shown in Figure 3B) is that in which expectation occurs earlier than the reward, and the drift rate w toward the observed interval can be corrected using Equations (5) and (6) to reduce the slope of the accumulator since ϕ reaches one. The rate change for that trial Δw(n) is accumulated until the next reward occurs.

(5) $$\begin{array}{r} \Delta \text{w}\left(\text{n},\text{t}\right)=\Delta \text{w}\left(\text{n},\text{t}-1\right)-{\left(\text{w}\left(\text{n}\right)+\Delta \text{w}\left(\text{n},\text{t}-1\right)\right)}^{2}\Delta t \end{array}$$

(6) $$\begin{array}{r} \text{w}\left(\text{n}+1\right)=\text{w}\left(\text{n}\right)+\alpha △\text{w}\left(\text{n}\right) \end{array}$$

where n denotes the number of training experiments, and α is the learning rate.

In the original paper (Rivest and Bengio, 2011), the above model was considered relatively simple and abstract. In order to apply it to our spiking neural network, we assume that the semaphore ϕ in the TDDM was the activity variable (*V*_*i*_) of the spiking neuron in Equation (2), thus establishing Equation (2) and (3), which is as follows:

(7) $$\begin{array}{r} \gamma_{m}\left(w\Delta t+\varepsilon \left(t\right)\right)=-V_{i}+I_{ext,i}+\overset{N}{\underset{j=1}{\sum }}L_{ij}V_{j} \end{array}$$

According to the above equation, the relationship between the drift rate and the weight of neural connections can be established. Therefore, the weights of the spiking neuron can be updated via the drift-rate-updating method described above in Equation (4) and (6).

In summary, the TDDM is a fast-learning model, and due to its drift feature, we can apply it to simulate ramp activity. The modified TDDM algorithm, like any other synaptic plasticity algorithm, can be explained as being affected by various neurotransmitters in the process of neuronal firing, thus dynamically adjusting synaptic weights.

There exists some other models to simulate ramp activity. Simen's work (Simen et al., 2011) has proposed that a specific form of diffusion model arises from simple assumptions about neural integration to achieve ramp activity. In this study, the model incorporates a rapid duration-learning procedure and accounts for a variety of physiological and behavioral finding by the diffusion model. The ideas of this research are similar to ours. However, there are substantial differences in the details between our work and their work. The form of the individual neuron model for our work is the ordinary differential equation, but for theirs is non-homogeneous Poisson spike generator. In addition, their diffusion model of interval timing is established at a high level, while ours is located in the connection between neurons.

### Neural-Circuit Model

Our neural circuit model was designed based on previous study (Merchant et al., 2011). In that study, electrophysiological SCT experiments were performed, in which five types of time-related cells were discovered in the MPCs of rhesus macaques, including motor cells, swing cells, relative-timing cells, absolute-timing cells, and time-accumulator cells. An obvious feature of these cells is ramp activity. It can be seen from the discharge rate diagram of the various neuronal types presented in the right column of **Figure 10** that all neurons—with exception of motor cells—will change their type of activity when changing the target interval. It can be observed that these cell types are all involved in the task of temporary processing. The conclusions from the Merchant et al. paper can be summarized as follows:

Relative-timing cells display monotonically rising ramp activity characteristics after time measurement begins. When they reach the threshold value, they will cause motor control, and then rapidly decline.

- Relative-timing cells interact with absolute-timing cells and their activity becomes locked at some point, resulting in a balanced loop mechanism for executing motor sequences with tight time structures.
- Neuronal activity of an absolute-timing cell exhibits an increase in its up-down profile of activation across different intervals. And they found that the duration of the up-down cycle of activity in absolute timing cells is associated with subjective time.
- The discharge diagram of a time-accumulator cell is similar to that of absolute-timing cells which represents the passage of time since the previous movement. And in time-accumulator cells, there is an additional increase in peak magnitude as a function of elapsed time. Thus, their slopes are similar across different target time intervals.
- As the target interval increases, the discharge period of swing cells increases. In addition the firing rates of swing cells always decrease and then increase within a target interval. We consider that the effect of swing cells may be to represent the interval length.

According to the above points, we assume that the entire neural circuit in the brain has the following time-processing procedure. First, absolute-timing cells are activated by the synchronization signal, and then the pulse-signal is simultaneously issued by the absolute-timing groups of various time scales. Next, due to the impetus of the absolute-timing cells, ramp activity of the relative-timing, and time-accumulator cells begins. In the meantime, in order to learn and reproduce the time interval, some synaptic plasticity (like the TDDM) is required in the connections between the absolute-timing and relative-timing cells. Finally, swing cells can represent the interval length through the learned interval from the relative-timing cells.

**Figure 4:**
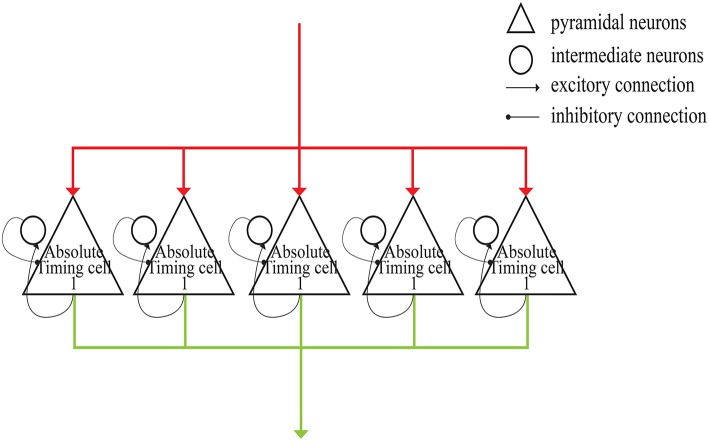
Structure of absolute-timing groups. The pyramidal neurons in different groups can last for different interval durations.

As shown in Figure 4, each absolute-timing group receives external stimuli simultaneously, with the differences among groups consisting of the weights of the excitatory and inhibitory connections between pyramidal neurons and interneurons. By setting various weights for different absolute-timing groups the firing rate of pyramidal neurons in each group can exhibit discharge curves similar to those found in a previous study (Merchant et al., 2011). In our design, the absolute-timing groups spanned different interval durations, which were not affected by the target interval.

**Figure 5:**
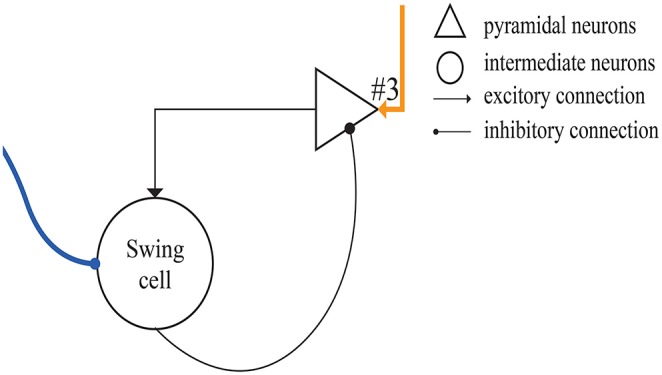
The microcircuit for swing cells which represent time interval.

Figure 5 shows the microcircuit we designed for swing cells based on the summary above. In Merchant's paper, there is little information about swing cells, leaving us to infer that the effect of swing cells may be to explicitly represent the interval duration on the basis of the discharge curve measured from them in the electrophysiological experiments. From the right column of **Figure 10D**, a period can be represented by the decrease and increase of the firing rate. That is, the high firing rate represents the start and the end of the duration. Similarly, the microcircuit is composed of a pyramidal neuron and an interneuron which is called swing cell. In the discharge diagram of swing cells, the firing rates decrease after the tap with lower and lower slopes and then increase when excitability becomes stronger than inhibition. Thus, the microcircuit receives two inputs, one (the blue line) from resetting cells and the other (the orange line) from time-accumulator cells. After tapping, resetting cells are activated to inhibit swing cells. At the same time, time-accumulator cells are also activated to excite the pyramidal neurons. As time passes, the inhibitory effect becomes weaker and the excitatory effect becomes stronger.

**Figure 6:**
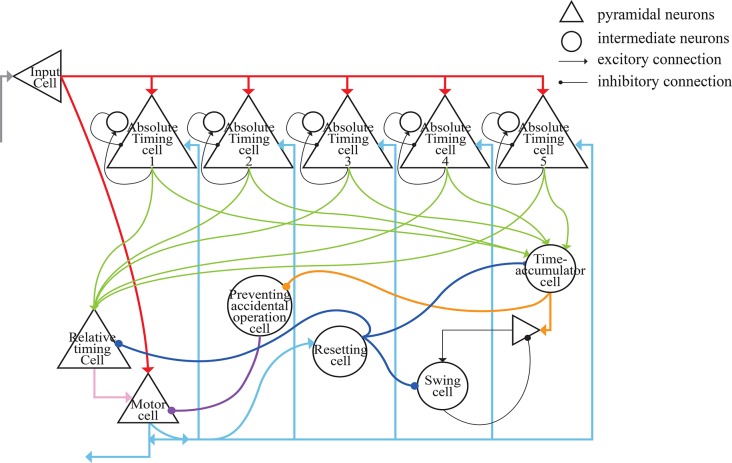
Architecture of the neural circuit in this study. In the figure, triangles represent pyramidal cells and circles are interneurons. These neurons, with the exception of relative-timing cells, were all simulated using the Izhikevich neuron model.

The architecture of our model is presented in Figure 6. The gray lines represent the input of external light or sound stimuli as synchronous signals to the input cells which lasted for only 100 ms. The red lines are the output connections from input cells. The input cells simultaneously activate absolute-timing cells that characterize different time intervals, and each absolute-timing group forms a microcircuit with interneurons (Figure 4), so that the slope of the ramp activity in each absolute-timing group is different. Since the motor cells perform a tap each time the input cells receive a synchronous stimulus during the synchronization phase of the SCT, there are connections between the input cells and the motor cells, as represented by the red lines. The green lines show that each group of absolute-timing cells simultaneously transmits spikes to the relative-timing cells, such that their firing rate continues to rise. As summarized above, there are also connections between absolute-timing cells and time-accumulator cells due to the ramp activity of time-accumulator cells which is similar to that of relative-timing cells. In order to make their slopes the same and their peaks rise as the target interval increases, the synaptic connections between the absolute-timing and time-accumulator cells must be different from those between the absolute-timing and relative-timing cells. The orange line shows the accident preventing operation cell we set up in order to prevent the motor cells from being activated prematurely by the connection represented by the purple line. Since the firing rate of swing cells has a down-up form, we constructed a microcircuit with the pyramidal neurons. When tapped, they receive the inhibitory stimulus of the resetting cells, and the discharge rate decreases. As time passes, the enhancement of the excitatory stimulation of the pyramidal cells in the microcell circuit leads to an increase in the firing rate of the swing cells, and the slope becomes less steep as it approaches the target interval. The pyramidal cells in the microcircuit receive the stimuli from the time-accumulator cells. The pink line shows that there is a threshold for the relative-timing cells. When the ramp activity reaches this threshold, we hypothesize that it is the achievement of the expectation time that causes the relative-timing cell to activate the motor cell, causing a tapping action. The light blue lines show that during the continuous phase of the SCT, since the external synchronous stimulus no longer exists, the motor cells need to act as synchronous signals via their connections with the absolute-timing cells. Meanwhile, in order to restart the period, the resetting cell—which is connected with the relative-timing, time-accumulator, and swing cells (represented by the blue lines)—should be activated by the motor cell.

The relationship between our model and the TDDM can be easily observed from the connection between the relative-timing and absolute-timing cells. As concluded above, for different target time intervals, the discharge rates of relative-timing cells will peak at different slopes. The process of increasing the firing rate corresponds to the process of accumulating evidence of time passing in the TDDM. Using the TDDM, we can achieve a kind of synaptic plasticity learning to fit the duration between the start and end of the ramp activity to the target interval time. Here, we have made some improvements to the TDDM. Compared with the original TDDM, the firing rate of the relative-timing cells can be regarded as the semaphore ϕ(t). Therefore, the peak of ϕ(t) is no longer one but is now the threshold of the firing rate. The initial weights between absolute-timing cells and relative-timing cells are set to the appropriate values. When the firing rate of relative-timing cells reaches the threshold earlier than the next synchronous signals, the weights will be tuned using TDDM until the next synchronous signals appear. Similarly, when the firing rate comes to the threshold later than the next synchronous signals, the weights will be immediately corrected using TDDM. Thus, our model can transform the collection of evidence of time passing into the accumulation of the firing rate by the synaptic weight.

## Computational Simulation Results

With the SCT experiments, we realized the learning of the time interval via the neural circuit we designed and we reproduced the discharge patterns of various neuronal groups described previously (Merchant et al., 2011).

In our experiments, we examined three time intervals— 400, 500, and 600 ms—and set the threshold of the relative-timing cells to 20 Hz. We considered an SCT experiment to be a training process in which there was a synchronous and a continuous phase. There were four taps during the synchronization phase and we made adjustments to the synaptic weights in the circuit three times (applying the TDDM algorithm). The continuous phase was based on all of the previous weight adjustments, and the tapping of the time interval to be learned was reproduced when there was no external synchronization-signal stimulus.

**Figure 7:**
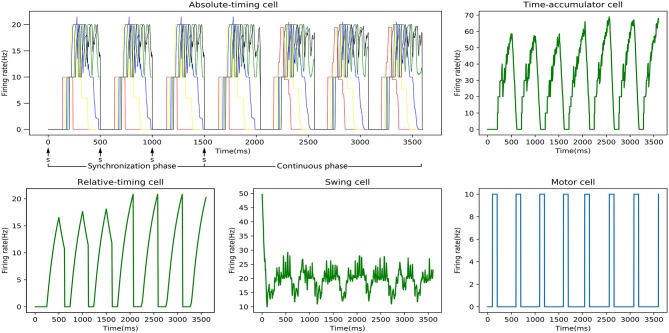
Discharge-rate curves of all neuronal types for the 500 ms experiment in an SCT time interval with our model. In the absolute-timing cell diagram, there are five firing rate curves of the pyramidal neurons in five absolute-timing groups. Due to the stimulus signal, the peak firing rate of the relative-timing cell in the synchronous phase that is lower than that in the continuous phase. The discharge rate of the swing cell represents a cyclical change from down to up. The motor cell was activated either after the external synchronization signals appeared or the firing rate of the relative cells reached the threshold.

One SCT experiment was one training process, and the discharge rate curve of each neuronal type in one experiment is shown in Figure 7. It can be seen that the relative-timing cells did not reach the threshold at the target time when a tap was performed in the synchronization phase, and the synaptic-weight adjustment was carried out three times in order to make the discharge rate closer to the threshold of 20 Hz. Obviously, the implementation of our synaptic-learning algorithm was beneficial to our time-learning model. After several training processes, the synaptic weights will stabilize within a certain range. The duration from a firing rate of 0 to the firing-rate peak of the relative-timing cell was our target duration. It is reasonable that there was a delay between the activation time of the motor cell and the time at which either the external stimulus appeared or the firing rate of relative cells reached threshold.

**Figure 8:**
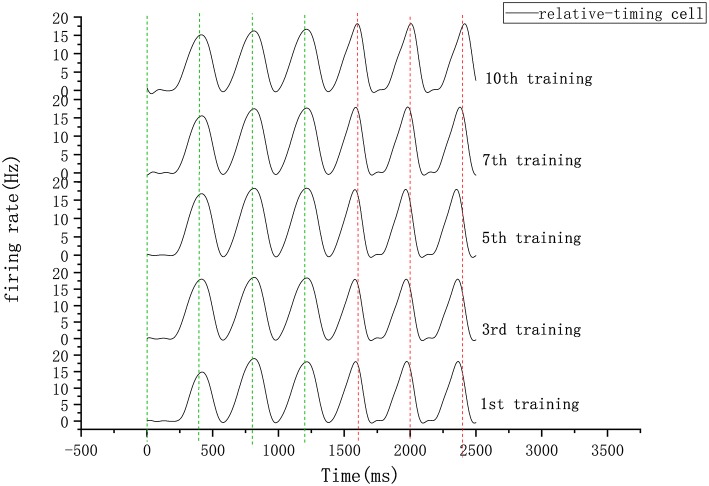
Firing rate diagram (after smoothing) of relative-timing cells during several training sessions. Here the interval length is 400 ms. The green-dashed lines represent the occurrence of external synchronization signals and the red-dashed lines indicate the desired tapping time points in the continuous phase.

**Figure 9:**
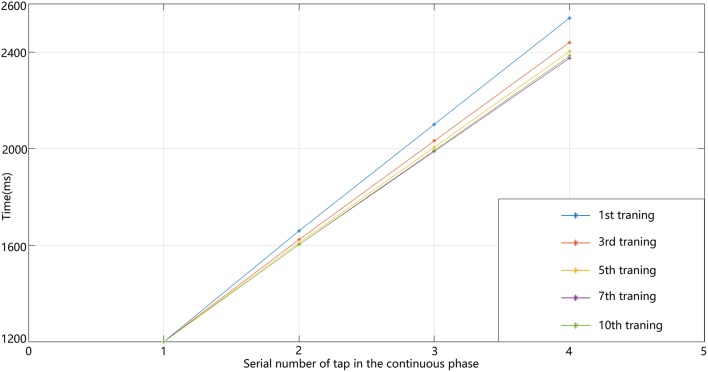
Line graph of four taps times in the continuous phase of the 400 ms experiment.

In our circuit, the relative-timing cell is considered to be the key to indicating the desired interval. The periodicity exhibited by each of the other neuronal groups is driven by the relative-timing cells. The discharge diagram of the relative-timing cells during several training sessions is shown in Figure 8. As the number of training sessions increase, the peak of the relative timing cells gets closer to the desired tap moment (red-dashed line). Figure 9 is a plot of the times the model learned. In the figure, the points of the continuous phase of each training session have been fitted with a straight line. It can be observed that our model's ability to learn the interval duration improved.

**Figure 10:**
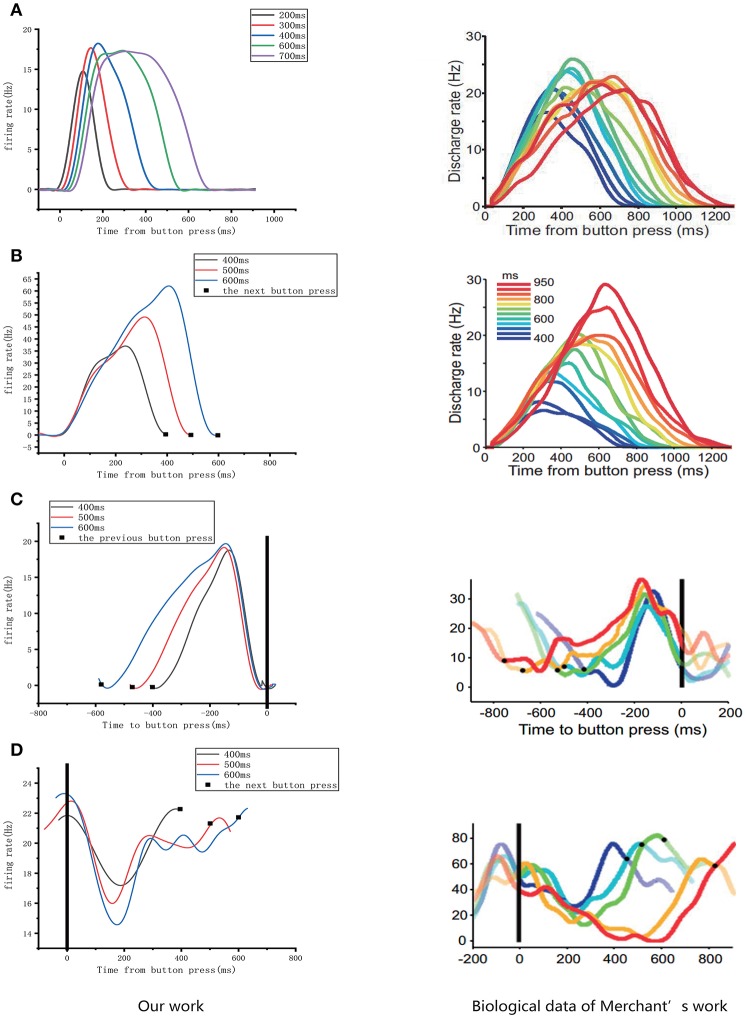
Comparison of the firing rates of all of the neurons in our model with results from electrophysiological measurements (Merchant et al., 2011). The left column is the discharge curve (smoothed) of the neurons in our model. The right column shows the discharge curves for all types of neurons from electrophysiological experiments. **(A–D)** respectively, illustrate the results for absolute-timing cells, time-accumulator cells, relative-timing cells, and swing cells (The right column of the figure is modified from Merchant et al., 2011).

As mentioned above, four types of cells related to time processing have been described previously (Merchant et al., 2011). These four types of neurons also exist in the neural circuits we designed. We compared them with the electrophysiological measurements of the firing patterns of neurons as shown in Figure 10. It is obvious that the neurons in our neural circuits exhibit periodicity and produce discharge curves similar to those observed in physiological experiments. In the left column of Figure 10A, the curves represent the firing rate of five absolute-timing groups which increase with different climbing rates for each color and span different time intervals. Similar to the right column of Figure 10B, the time-accumulator cells we designed increased with rising at the same climbing rates as their electrophysiological counterparts, although the peaks differed for different time intervals. In order to be consistent with the discharge rate diagram of relative-timing cells in the reference, the figure in the left column of Figure 10C was plotted in the same form. It can be seen that our relative-timing cells exhibited the same features as those in the discharge rate diagram. Figure 10D shows the firing rates of the swing cells in our model and those of the electrophysiological experiment. Although there are some differences in the curves of the two graphs of Figure 10D, our swing cells retained the characteristics of the bio-cell discharge rate. One period of the neurons we designed in the left column of Figure 10D can represent the target-time interval; the amplitude of the curve increases as the target interval duration increases. We believe that the reason for the difference between the curves of our experimental results and those of electrophysiological experiments has to do with the fact that our results were somewhat smoothed and also that the time-window selection used to calculate the discharge rate of neurons in our model differed from that used in the electrophysiological experiments. Therefore, we consider that the differences observed in the curves of Figure 10D are acceptable.

**Algorithm 1:** The flow of our models which interprets the architecture shown in Figure 6.

1:	Initialize: S ← the parameters of all spiking neurons;
	W ← the weights of each other neuron in our neural circuit;
	T ← An array which contains the time when the external stimulus begins
	to appear;
	r ← drift rate;
	t ← time(ms)
	*I*_external_ ← the current of external stimulus;
	*V*_threshold_ ← the firing rate threshold of relative-timing cells for TDDM
	*V*_relative_ ← the firing rate of relative-timing cells
2:	** while** t>0 **do**
3:	**if** t in synchronous phase **then**
4:	**if** t in T **then**
5:	*I*_external_ stimulates the input cells for only 100 ms(gray line)
6:	**if** t! = 0 AND t in T **then**
7:	**if** *V*_relative_ < V_threshold_ **then**
8:	Implement the Equations 4 and 6 to modify r.
9:	**else**
10:	Implement the equation 6 to modify r.
11:	**end if**
12:	And then according to equation 7 update w in the connections between absolute-timing cells and relative-timing cells
13:	**end if**
14:	**else**
15:	**if** *V*_relative_ > *V*_threshold_ **then**
16:	Implement the equation 5 to obtain Δr:
17:	**end if**
18:	**end if**
19:	**end if**
20:	According to the architecture in Figure 6, compute all the neurons.
21:	Compute the state of the input cells using equation 1.
22:	Compute the state of absolute-timing cells using equation 1.
23:	Compute the state of the time-accumulator cells using equation 1.
24:	Compute the state of the relative-timing cells using equation 2.
25:	Compute the state of the motor cells using equation 1.
26:	Compute the state of the other cells using equation 1.
27:	**end while**

**Figure 11:**
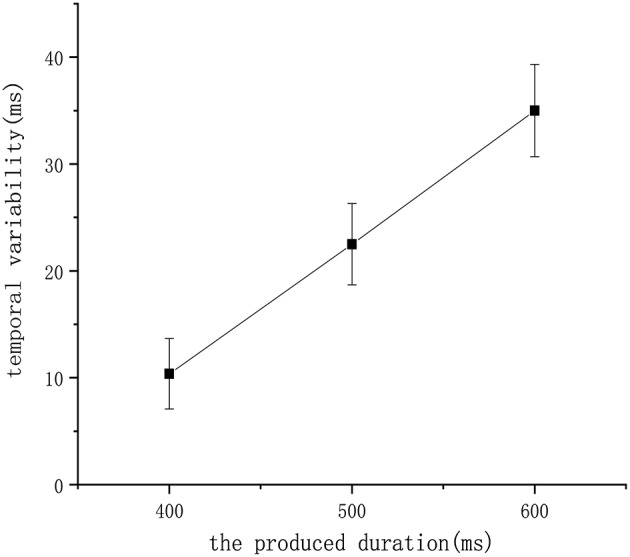
The relationship between temporal variability and the produced duration in our model.

Finally, we tested our model to check whether it satisfies an additional biological property called the scalar property (Gibbon et al., 1997), which tells us that the uncertainty is proportional to the interval being estimated. This property has been interpreted to indicate that the variability of an underlying temporal distribution should exhibit a constant coefficient of variation (σ/μ). Figure 11 shows that repeated the experiment eight times and recorded the mean of the learned duration in the 400 ms experiment, 500 ms experiment, and 600 ms experiment receptively. According to the figure, the scalar property was followed by our model, which further confirms the biological interpretability of our work.

## Conclusion and Discussion

In this study, in order to explore a possible time-processing mechanism in the human brain, we examined the time processing behind the electrophysiological phenomena observed in the SCT experiment (Merchant et al., 2011), and we presented a new neural circuit based on specific neural-connection structures utilizing a TDDM algorithm as the synaptic learning mechanism. This neural circuit was successful in determining time intervals in SCT experiments and in expressing the time intervals learned, indicating that our proposed method is reasonable and effective. Although computational simulation results— which are often more idealized— tend to differ from those of electrophysiological experiments, our simulation experiments and physiological test results were completely consistent with those of electrophysiological experiments. This suggests that the circuit we designed is similar to the endogenous circuit of the macaque brain in terms of achieving this particular timing and periodicity operation.

**Figure 12:**
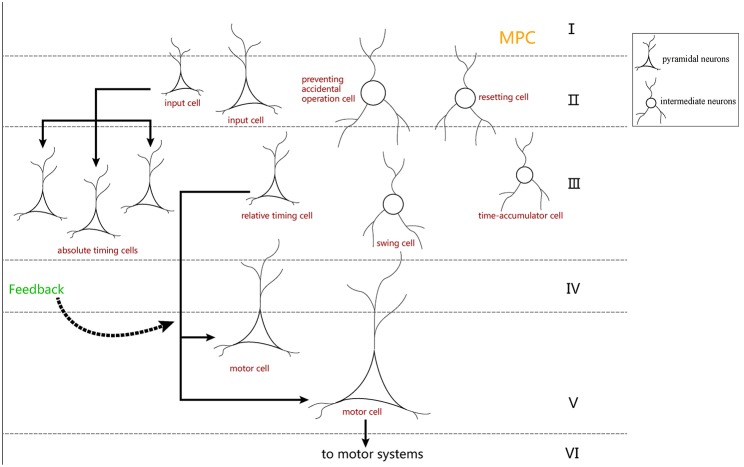
The distribution hypothesis of our neuron-circuit model. We hypothesize that the input cells used as the cue cells should reside in the superficial layers which receive the stimuli from the outside environment. Additionally, the motor cells used as the output cell are suitable for layer V, where the cells transmit out signals for movement. Finally, layer III is always used to integrate the information, so the four kinds of ramp cells should reside in layer III.

In neurobiology, the cerebral cortex can be divided into different regions according to different functions. The hierarchical structure of each brain region is essentially the same, and it is composed of six layers of neurons: molecular layer, external granular layer, external pyramidal layer, internal granular layer, internal pyramidal layer, and multiform layer (Le Be', 2007). These six layers of neurons are arranged vertically in each brain region. According to previous literature (Merchant et al., 2014), it is known that MPC is more active in SCT experiments. Additionally, there are many time-related cells in the MPC. We believe that the neurons in the neural circuit we designed may also be distributed in the six layers of the MPC (Figure 12).

The most significant difference between our model and previous simulations is that our model is more biologically interpretable. Compared with the LSTM model (Rivest et al., 2010), the drift diffusion model during the SCT (Merchant and Averbeck, 2017), and time-adaptive drift–diffusion model (Rivest and Bengio, 2011), spiking neuron which models the real biological neurons are used in our model. And Recreating the discharge rate curves observed and scalar property in electrophysiology is another advantage of our model compared with pacemaker accumulator models (Treisman, 1963) and state dependent network models (Buonomano and Maass, 2009). The downside of our model is that it can only learn very precise interval durations. In our model, we make use of TDDM algorithm, which is considered to be a fast and accurate time-learning mechanism for determining interval durations. Although humans can learn target time, there must exist some biases between the time learned and target time. Hence, the high accuracy which is the advantage of TDDM algorithm cannot be interpreted biologically. A certain degree of biases are necessarily presented in human experiments. We believe our model can become more biologically plausible by adjusting the parameters of TDDM algorithm such as ε(t).

Knudsen et al. (2014) found the same four types of ramping cells in the primary motor cortex in a single interval reproduction task in rats. Their work provided us more information to improve our model in the future. In addition, we will improve our learning-mechanism algorithm to satisfy additional biological properties and be flexible in more temporal-processing experiments. And now this work is based on the property called ramp activity, but future studies will explore other properties as well. We believe that as more and more time-related biological properties are adopted, our model will become closer to endogenous biological processing mechanisms.

## Author Contributions

HW carried out the computational neuroscience model and neural circuit design studies, experiment design, and writing the paper. Y-FD designed algorithms, coded simulation program and collected experimental data, and wrote the paper. All authors read and approved the final manuscript.

### Conflict of Interest Statement

The authors declare that the research was conducted in the absence of any commercial or financial relationships that could be construed as a potential conflict of interest.
